# Two-stage scheduling optimization model for taxiways on the basis of time slot derivation

**DOI:** 10.1371/journal.pone.0345176

**Published:** 2026-03-20

**Authors:** Jiyu Tang, Qigui Zhang, Guan Lian, Tao Wen

**Affiliations:** 1 Guangxi Airport Management Group Co., Lid, Nanning, China; 2 Guangxi Key Laboratory of Intelligent Transportation, Guilin University of Electronic Technology, Guilin, China; 3 The Second Research institute of Civil Aviation Administration of china, Chengdu, China; Southwest Jiaotong University, CHINA

## Abstract

The continuous growth in air traffic at civil airports has placed significant economic pressure on surface operations. Consequently, strategic adjustments to departure schedules and optimization of taxiing routes have become essential to reduce operational costs. This study proposes a two-stage optimization framework aimed at minimizing surface operation expenditures. In the first stage, a dynamic pushback slot control (DPSC) strategy is employed to regulate departure sequences. The second stage enables preplanning of taxi routes for both arriving and departing aircraft by optimizing the taxiway control threshold, thereby refining the pushback slots for departing flights. To support route planning, multiple taxiing configurations are generated for different departure intervals. To improve solution quality and mitigate ground conflicts, an improved ant colony algorithm (IACA) incorporating a negative feedback mechanism is developed. Experimental results show that, compared to a baseline scenario without departure control, the proposed framework reduces taxiing costs by 17.8%, yielding an optimized total cost of USD 8,163.44. Furthermore, relative to strategies without the negative feedback mechanism, the proposed approach achieves an average cost saving of USD 1,412.71. These results demonstrate that the proposed framework provides superior economic benefits while simultaneously improving operational safety and efficiency.

## Introduction

Taxiway congestion and operational conflicts at large-scale, high-density airports constitute significant bottlenecks for airport throughput and lead to excessive fuel consumption. Statistics indicate that global aviation fuel consumption has surpassed 5 million barrels per day since 2013; such high consumption levels inevitably result in elevated pollutant emissions [1]. Furthermore, aircraft surface movement within the maneuvering area currently remains heavily reliant on manual sequencing and scheduling. As traffic volume escalates—particularly during peak hours or adverse weather conditions—the complexity of taxiway management intensifies, with human factors contributing to approximately 75% of aviation incidents [2]. Conventional operational control methods, which necessitate high levels of inter-personnel coordination, are increasingly characterized by inefficiency and a heightened risk of ground accidents, potentially leading to aircraft damage or even casualties.

Therefore, in the preplanning stage of aircraft taxi route optimization, taxiway queuing time can be transformed into parking stand waiting time by regulating the frequency of aircraft pushback. This approach effectively alleviates surface taxi congestion and enables a dynamic balance between taxiing efficiency and fuel consumption.

To mitigate airport surface taxi congestion, numerous operational strategies have been proposed and implemented in practice. Pushback control is a commonly adopted approach, which prevents excessive taxiway congestion by regulating the timing of aircraft pushback from gates. In contrast, taxiway optimization focuses on applying intelligent algorithms to design the shortest or most efficient taxi routes for individual aircraft, thereby reducing taxiing time and fuel consumption. When pushback control strategies are employed, it is essential to appropriately balance the costs associated with parking stand waiting, taxiway fuel consumption, and taxiing conflicts. Achieving this balance is critical for reducing overall surface operation costs and improving airport operational efficiency.

The issue of aircraft taxi scheduling on airport surfaces has been extensively studied by domestic and international scholars, who have focused on various perspectives, such as modeling approaches and solution techniques. In terms of modeling methods, Rathinam et al. [3] constructed a mixed integer planning model by adding constraints such as interaircraft wake spacing to improve the applicability of the model. Deau et al. [4] proposed a concept at the airport operation control level to address the issue of taxi sequences and runway occupancy sequences for approaching and departing aircraft and to plan taxi sequences and routes for approaching and departing flights at the preplanning stage. Roling et al. [5] proposed a mixed-integer planning model considering multiple alternative paths, aiming to improve the robustness of the model under uncertain inbound and outbound times, and verified the feasibility of the model in a simulation environment. Anderson et al. [6] considered the constraints of aircraft crossing taxiways and constructed a robust combined integer linear programming model. Their approach addressed the complexities of aircraft ground movements while maintaining operational efficiency. Guépet et al. [7] evaluated aircraft taxi times and completion times as performance indicators while also considering flight punctuality. They verified the model’s reliability via real-world data and demonstrated the conflicting relationship between aircraft taxi duration and pollution emission indicators. Park et al. [8] developed a model to predict taxi times at airports by considering various influential factors, such as airport weather, the number of take-off and landing flights, the number of aircraft moving in the moving area, the runway occupation time for arrival, the runway occupation time for departure, the take-off and landing directions, and the terminal location. The goal is to optimize aircraft flow while accounting for weather and other relevant variables. Soltani et al. [9] proposed a hybrid taxiing solution to reduce airports’ impact on GGEs, where part of the taxiing operations is handled by two trucks powered by renewable energy while other aircraft continue using their engines to complete taxiing. They primarily addressed how to integrate collision avoidance mechanisms into hybrid taxiing systems at airports while optimizing fuel consumption and service quality, thereby enhancing the safety and efficiency of taxiing operations.Gao et al. [10] developed a two-phase optimization model that integrates aircraft stand assignment with tow-tractor routing to minimize operational fuel consumption at airports, achieving approximately 5% reduction in fuel usage. The study employed an improved NSGA-II algorithm that distinguishes ground vehicle scheduling based on task attributes while balancing multiple stakeholder interests including passenger satisfaction and operational efficiency.Yan et al. [11] proposed a chance-constrained optimization model for pre-departure flight scheduling that addresses capacity uncertainty in both airports and sectors at the national air traffic network level, incorporating node importance metrics to set violation probabilities. Their approach, validated using real flight data from 3,435 flights across 195 airports and 188 sectors, demonstrates that considering network structural characteristics produces more robust flight schedules with lower total costs compared to traditional deterministic or uniform probability models.Fan et al. [12] developed a slot allocation optimization model for Multiple Airport Systems (MAS) that simultaneously considers airport capacity and airspace fix capacity constraints, addressing the multi-resource allocation challenge where airlines compete for both airport slots and shared airspace resources.

In terms of algorithmic solutions,Weiszer et al. [13] used a multiobjective route and scheduling algorithm AMOA * for airport ground motion, which considers the real speed curve and finds the optimal or near-optimal route for a fixed sequence of aircraft on the basis of taxi time and fuel consumption. Maadanpour et al. [14] established a triobjective mathematical model for the transportation-location-routing problem. Brownlee et al. [15] proposed an adaptive Mamdani fuzzy rule-based system to estimate taxi times and their uncertainties. Furthermore, the existing quickest path problem with time windows (QPPTW) algorithm is adapted to use fuzzy taxi time estimates. Sekine et al. [16] proposed a scientific system design for operationally feasible AMAN assisting air traffic controllers (ATCos) through runway-flow and interaircraft control. They devised an airline-oriented runway assignment rule that selects a target minimizing the arrival taxi time in the case of overdemand according to the maximum estimated through the stochastic distributions of the interaircraft time and runway occupancy time. Benlic et al. [17] constructed an aircraft taxi scheduling model for large airports on the basis of a space-time network and introduced distance evaluation coefficients and transition evaluation coefficients to optimize paths. Katsigiannis et al. [18] proposed a new triobjective slot allocation model (TOSAM) that considers total schedule displacement, maximum schedule displacement, and demand-based fairness, and they introduced a multilevel, multiobjective algorithm to solve it. They used real-world slot requests and airport capacity data to demonstrate the applicability of the proposed approach. Ahmadian et al. [19] developed an efficient matheuristic algorithm for the aircraft landing problem (ALP). The ALP aims to schedule aircraft landings such that the total deviation from target arrival times is minimized. Otmane et al. [20] used a tactical planning tool: it consists of assigning efficient and nonstop routes to the scheduled traffic on departure. This tool, which uses a real-time algorithm, detects routing conflicts in advance and solves them before aircraft leave stands and approaches hotspots in the taxing network.Ravizza et al. [21] adopted a cost conversion strategy, converting aircraft delay time on taxiways into waiting time at stands to reduce aircraft taxiing costs. Goncharenko et al. [22] developed a simulation program to analyze various dispatching algorithms for aircraft operations. The program tracks the number of free requests and the status of available and occupied aircraft at each step. Feuser et al. [23] proposed a noniterative real-time model to minimize the waiting time in runway queues. This model can assist air traffic controllers in making decisions during ground congestion at airports. Bao et al. [24] proposes a novel dynamic airport ground movement optimization framework that incorporates refined link-level unimpeded taxi time estimation, data-driven conflict priority assignment, and adaptive traffic situation awareness to address taxiing uncertainties and generate real-time conflict-free trajectories. Wang et al. [25] developed a data-driven approach for determining airport declared capacity that addresses two critical challenges: operational uncertainty across scheduling seasons and the balance between slot creation benefits and congestion costs. Desai et al. [26] proposed a penalty-based dynamic departure pushback control (PDPC) strategy that uses a linear penalty function based on taxiway queue limits and current queue lengths to manage the pushback frequency at airports. The strategy aims to minimize total operational costs by balancing taxiway queueing times with gate-hold delays.Wang et al. [27] addressed the slot allocation problem for Multiple Airport Systems (MAS) by developing a chance-constrained optimization model that simultaneously considers both airport capacity and critical airspace fix capacity constraints while accounting for flying time uncertainty between airports and fixes.

A synthesis of existing studies indicates that most current research concentrates on optimizing isolated components of airport surface operations, such as taxi route selection or taxi time prediction, while largely treating pushback control and taxiway congestion management as independent problems. In practice, however, airport surface operations form a continuous and highly coupled system. If gate pushback scheduling and inbound–outbound traffic flows are not jointly considered during the planning process, taxi route optimization alone is often constrained by taxiway topology or capacity bottlenecks, leading to solutions that deviate from actual operational conditions. Moreover, relatively little attention has been given to the dynamic interactions among taxiway queuing thresholds, pushback frequency regulation, and the mechanisms driving conflict formation during aircraft taxiing.

To address the above issues, this paper proposes a two-stage airport surface operation optimization framework that integrates dynamic pushback control with conflict-aware taxi path planning. In the first stage, a taxiway queuing threshold–based pushback control model is developed to regulate aircraft pushback frequency, thereby mitigating excessive congestion in the taxiway network at its source and converting high-cost taxiway waiting time into lower-cost gate-hold waiting time. In the second stage, based on the outcomes of the pushback control model, a taxi path planning model is established that explicitly considers aircraft priority and conflict avoidance. To efficiently solve the proposed optimization problem, an improved ant colony algorithm is designed to reduce taxiing conflicts while simultaneously balancing operational efficiency and fuel consumption.

The main contributions of this study are summarized as follows:

(1) A dynamic pushback control model based on taxiway queuing thresholds is proposed to regulate the inflow of aircraft into the taxiway system, thereby mitigating airport surface congestion at its source.
(2) Under the constraints imposed by the pushback control strategy, a conflict-aware taxi path planning model is developed that accounts for aircraft priority and operational interactions.
(3) An improved ant colony algorithm is designed to efficiently solve the integrated optimization problem, achieving a simultaneous reduction in taxiing conflicts and airport surface operating costs.
(4) The effectiveness of the proposed approach is validated through a real-world airport case study, and its performance advantages are demonstrated via comparative analysis.

The remainder of this paper is organized as follows. Section 2 reviews the related literature and summarizes the main approaches for improving airport operational efficiency. In Section 3, a gate-hold waiting model incorporating dynamic pushback control is proposed to determine aircraft pushback times under different queuing thresholds. Based on the pushback control model, a taxi route planning model is then developed, and an improved ant colony algorithm (IACA) that accounts for flight priority and conflict avoidance is designed. Case studies and numerical analyses are presented in Section 4 to evaluate the proposed approach, including comparisons with strategies without conflict control and an analysis of optimal taxiing costs. Finally, Section 5 concludes the paper and outlines directions for future research.

## Dynamic optimization model for flight taxiing paths with negative feedback constraints

### Description of the problem

The airport surface aircraft path planning problem aims to assign conflict-free taxi routes to aircraft within specified dispatch time windows, thereby ensuring that take-off and landing operations can be completed as scheduled. In addition, minimizing aircraft taxiing time on the airport surface is a key objective, as it directly contributes to reducing airline operating costs.

### Model assumption

To determine departure aircraft pushback slots and model taxiing conflict avoidance, the following assumptions are adopted:

(1) To facilitate model solution, all aircraft are assumed to taxi at the same operating speed, and stopping or waiting during the taxiing process is not permitted. When a taxiing conflict occurs, it is resolved according to predefined priority rules, and each pair of aircraft is assumed to experience at most one conflict, with subsequent node conflicts being ignored. The taxi route of each aircraft is selected from a predefined set of feasible taxiing paths.
(2) Adjacent parking stands are aggregated into a single unified stand, and all arriving aircraft are assigned to the corresponding consolidated designated stand.
(3) An aircraft is assumed to enter the taxiway system immediately after pushback, and the distance traveled before entering the taxiway is neglected. Arriving aircraft are assumed to have priority access to the taxiway system, and the landing time is regarded as the time at which the aircraft enters the taxiway. The definitions of the notations are provided in Table 1.

**Table 1:** Notation and definitions used in this study.

Notation	Definition	Notation	Definition
$T_{f_{i}}^{A,S}\;$	Taxiing slot of inbound aircraft $f_{i}\;$	$G_{\max}\;$	Maximum allowable waiting time
$f_{i}\;$	Aircraft (indexed by *i*)	$T^{\text{taxi}}\;$	Average taxiing time
*ω*	Fuel consumption rate during parking	GAP	Percentage reduction in taxiing cost
*η*	Fuel consumption cost per unit idle time	$T_{\text{taxi}}^{*}\;$	Average taxiing time under threshold
$PAT_{f_{i}}\;$	Actual pushback time of aircraft $f_{i}\;$	$conf^{*}\;$	Average taxiing cost of aircraft
$POT_{f_{i}}\;$	Scheduled pushback request time	${\text{iter}}_{\max}\;$	Maximum number of iterations
$c_{2}\;$	Total taxiing cost of aircraft	$pri_{f_{i}}\;$	Priority level of aircraft
$T_{f_{i}}^{A}\;$, $T_{f_{i}}^{D}\;$	Total taxi times (arriving/departing)	$T_{k}^{\text{delay}}\;$	Total delay time
$x_{uv}\;$	Path determination factor	$con_{a}^{f_{ij}}\;$	Number of taxiing conflicts
$d_{uv}\;$	Distance between nodes $(N_{u},N_{v}$	*α*	Pheromone importance factor
$V_{f_{i}}\;$	Taxiing speed of aircraft	*β*	Heuristic information factor
$T_{f_{i}}\;$	Time to complete taxiing	*ρ*	Pheromone evaporation rate
		*Q*	Pheromone intensity constant

### Dynamic pushback slots control model

By controlling the frequency of aircraft pushback and rationalizing the pushback time of aircraft, the taxi waiting time can be reduced, the chance of delay can be reduced, and the overall punctuality of the airport can be improved. Generate the departure aircraft taxi start time via the Dynamic Pushback Control Model control strategy.


$$\begin{array}{c} \mu^{\prime }=\left\{ \begin{array}{cc} \mu \left(1-\frac{I}{L}\right) & I<L\\ 0 & I\geq L \end{array}\right.\; \end{array}$$ (1)

The first stage uses the departure aircraft pushback delay time as the optimization objective and converts the high-cost taxiing time into a low-cost parking space waiting time within the pushback time allowance, with the decision variables being the parking space queuing threshold and the inbound aircraft taxiing slot $T_{f_{i}}^{A,S}\;$. The objective function of the pushback slot optimization model is as follows:


$$\min c_{1}=\sum_{i=1}^{n}(PAT_{f_{i}}-POT_{f_{i}})\cdot \omega \cdot \eta \;$$ (2)

subject to the constraints:


$${\text{PAT}}_{f_{i}}-{\text{POT}}_{f_{i}}\leq 15,\;$$ (3)

$${\text{PAT}}_{f_{i}}\leq {\text{PAT}}_{f_{j}},\;$$ (4)

$${\text{DATS}}_{f_{i}}>{\text{PAT}}_{f_{i}}\;$$ (5)

where $f_{i}\;$ represents an aircraft pushback request, *ω* denotes the replacement coefficient for aircraft parking fuel consumption, *η* is the fuel consumption value per unit time of taxiing ($/min), $PAT_{f_{i}}\;$ indicates the actual pushback time of the departing aircraft, $POT_{f_{i}}\;$ represents the original pushback request time, and $DATS_{f_{i}}\;$ is the aircraft taxi start time. The Eq (3) limits the aircraft parking time to no more than 15 minutes. The Eq (4) ensures that a departing aircraft cannot overtake the preceding departing aircraft on the same runway before starting taxiing, thereby maintaining the pushback sequence. The Eq (5) requires that the departing aircraft cannot begin taxiing until after the actual pushback time. To ensure the efficient utilization of runways and taxiways and reduce queuing congestion during aircraft departure processes, airports typically control aircraft pushback rates. Converting taxi costs into delay costs can significantly reduce fuel consumption during aircraft taxiing. This paper aims to reduce aircraft ground operation costs by limiting the number of aircraft pushbacks and converting taxi costs into parking stand waiting costs within a delay time threshold. This approach effectively transforms surface movement costs into waiting costs at the gate. The exponential function $y=e^{at}-1\;$ is chosen as the cost transformation function.

The cost conversion factor is shown in Eq (6):


$$\alpha =\frac{\ln(yx+1)}{\;x}\;$$ (6)

**Fig 1:**
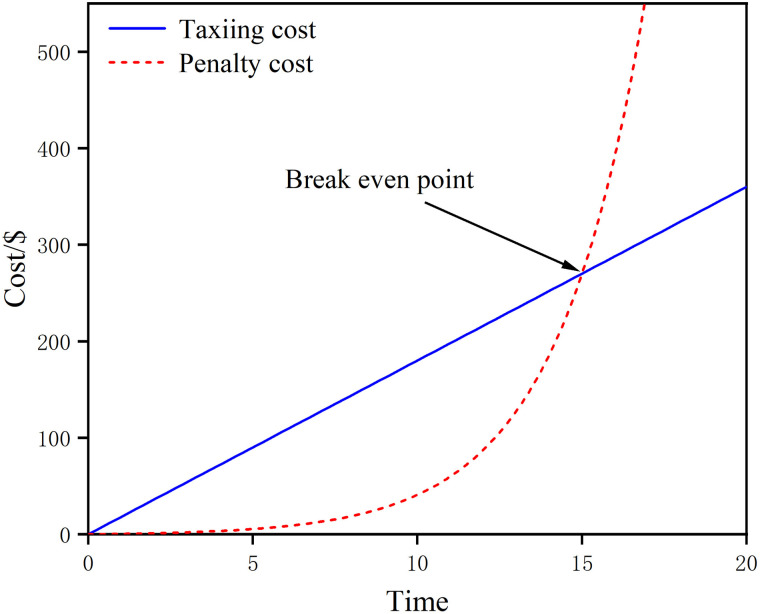
Fuel cost and conversion cost curves.


According to the relevant regulations of the airport, the actual take-off time within 0–15 minutes of the expected take-off time is a short delay. To reduce the adverse impact of aircraft occupying the parking space for a long time, this paper sets the threshold time as 15 minutes. The cost of fuel consumption is equal to the cost of waiting at the gate for 15 minutes, which is obtained by α=0.3734 . Therefore, the waiting cost incurred by the aircraft at the gate per unit of time condition is $y=e^{0.3734x}-1\;$. As shown in Fig 1, when the aircraft stopping time is less than 15 minutes, the stopping cost is significantly less than the taxiway taxiing cost, and when it is more than 15 minutes, the increasing trend of the stopping cost for waiting is obvious, which indicates that at this time, the aircraft should not be overstayed. Eq (2) can be transformed into Eq (7).

$$c_{1}=\min\sum_{i=1}^{n}\left(e^{\alpha (PAT_{f_{i}}-POT_{f_{i}})}-1\right)\;$$ (7)

### Path optimization model based on a negative feedback mechanism

To address the issue of the coordinated optimization of aircraft pushback times and taxi paths during surface operations, a two-stage optimization model is established. In the first stage, the optimization target is the aircraft’s taxiing start time. Considering potential conflicts during the taxiing phase and runway takeoff waiting issues, this stage opts to convert aircraft runway obstacle avoidance or runway waiting time into parking stand waiting time. By controlling the aircraft pushback frequency, the efficiency of taxiway resource utilization is improved. The second stage focuses on optimizing aircraft taxi paths. This is achieved by assigning appropriate taxi routes to all aircraft movements within the planning period. During the preplanning stage of the taxiing path, if the current aircraft’s taxiing route has a high number of conflicts with other aircraft, alternative routes are considered. In this process, the taxiing cost of the aircraft is also taken into account, ultimately generating a taxiing path for the current aircraft that balances both taxiing cost and conflict constraints.


$$minc_{2}=\left(\sum_{i=1}^{m}T_{f_{i}}^{A}+\sum_{j=1}^{n}T_{f_{j}}^{D}\right)\eta +\sigma \cdot \text{Con}\cdot \eta \;$$ (8)

$$DIS_{f_{i}}=\sum_{u=1}^{n}\sum_{v=1}^{n}x_{uv}d_{uv}\;$$ (9)

$$T_{f_{i}}=\frac{DIS_{f_{i}}}{v}\;$$ (10)

Eq (8) is the path planning objective function. $c_{2}\;$ is the total cost of taxiing an aircraft. $T_{f_{i}}^{A}\;$, $T_{f_{j}}^{D}\;$ are the total taxi times for incoming and outgoing aircraft, respectively. In Eq (9), $x_{uv}\;$ is the path determination factor. If aerial vehicle $f_{i}\;$ passes through node $(N_{u},N_{v})\;$, then $x_{uv}=1\;$; otherwise, $x_{uv}=0\;$. $d_{uv}\;$ is the distance between nodes $(N_{u},N_{v})\;$, and $\sum_{u=1}^{n}\sum_{v=1}^{n}x_{uv}d_{uv}\;$ is the total glide distance of the aircraft between the glide link nodes. In Eq (10), for ease of calculation, it is assumed that the aircraft glides along each link at a constant speed, where $V_{f_{i}}\;$ is the glide speed of aircraft $f_{i}\;$. $T_{f_{i}}\;$ is the time required for the aircraft to complete the taxiing phase. *σ* is the aircraft taxiing conflict penalty time factor, and Con  is the number of aircraft conflicts at each queuing threshold.


$$T^{\varphi }=T_{1,f_{i}}\;$$ (11)

$$\frac{d_{uv}\;z_{v_{1}v_{2}}^{f_{i}}}{V}-M(1-z_{v_{1}v_{2}})\leq t_{v_{1}}^{f_{i}}-t_{v_{2}}^{f_{i}}\;$$ (12)

$$(z_{v_{1}v_{2}}^{f_{1}}t_{v_{1}}^{f_{1}}-z_{v_{1}v_{2}}^{f_{2}}t_{v_{1}}^{f_{2}})(z_{v_{1}v_{2}}^{f_{1}}t_{v_{2}}^{f_{1}}-z_{v_{1}v_{2}}^{f_{2}}t_{v_{2}}^{f_{2}})\leq 0\;$$ (13)

$$(z_{v_{2}v_{1}}^{f_{1}}t_{v_{1}}^{f_{1}}-z_{v_{2}v_{1}}^{f_{2}}t_{v_{1}}^{f_{2}})(z_{v_{1}v_{2}}^{f_{1}}t_{v_{2}}^{f_{1}}-z_{v_{2}v_{1}}^{f_{2}}t_{v_{2}}^{f_{2}})\leq 0\;$$ (14)

$$|t_{v_{1}}^{f_{1}}-t_{v_{1}}^{f_{2}}|<\Delta t_{\text{safe}}\;$$ (15)

$z_{v_{2}}^{f_{i}}\;$ is the glide path node determination parameter; if the taxiing path of aircraft $f_{i}\;$ passes from node $v_{1}\;$ through node $v_{2}\;$, the value is 1; otherwise, it is 0. *t* is the time at which an aircraft passes through a particular node. *M* is a sufficiently large penalty factor. Eq (11) passes the aircraft taxi start time obtained from the pushback control phase to the path planning phase. Eq (12) indicates that the time difference between the arrival of an approaching and departing aircraft at any two connected nodes is greater than or equal to its taxi time between the two nodes, i.e., the continuity constraint of aircraft taxiing. Eq (13) is used to detect interaircraft cross conflicts. Eq (14) is used to detect tailing conflicts between any two aircraft. Eq (15) is used to detect head-to-head conflicts between two aircraft. Considering that different aircraft have different taxiing priorities, this paper designs priority constraints. The core idea is to assign a priority to each task or operation and decide the execution order of the tasks according to the priority level. In this work, a priority scheduling strategy is used to design a two-dimensional priority table for each aircraft by considering the aircraft type and the type of approach and departure, which is calculated as follows:


$${pri}_{f_{i}}(\alpha_{f_{i}},\beta_{f_{i}})=\frac{\left(\alpha_{f_{i}}+\beta_{f_{i}}-1)(\alpha_{f_{i}}+\beta_{f_{i}}-2\right)}{2}+\alpha_{f_{i}}\;$$ (16)

$$\begin{array}{c} \alpha_{f_{i}}=\left\{ \begin{array}{cc} 1 & f_{i}\text{ is a landing flight}\\ 2 & f_{i}\text{ is a departure flight} \end{array}\right.\; \end{array}$$

$$\begin{array}{c} \beta_{f_{i}}=\left\{ \begin{array}{cc} 1 & f_{i}\text{ is an H flight}\\ 2 & f_{i}\text{ is an L flight}\\ 3 & f_{i}\text{ is an S flight} \end{array}\right.\; \end{array}$$

where $pri_{f_{i}}\;$ is the aircraft priority; the smaller the value is, the higher the priority of the aircraft. The priority of each aircraft is shown in Table 2.

**Table 2:** Aircraft avoidance priorities.

*α*	*β*		
	H-shape	L-shape	S-shape
Landing aircraft	1	2	4
Departure Aircraft	3	5	8

The priority constraints are as follows:


$$P_{f_{i}}\leq P_{f_{j}}\;$$ (17)

Eq (17) is used for aircraft priority judgment; if the current aircraft priority is greater than the previous sequence of aircraft, the number of conflicts does not increase.

## Multi-aircraft path planning algorithm based on negative feedback

To address the challenges of calculating aircraft taxiing delay times and preplanning taxi routes under potential taxiing conflicts, the proposed algorithm comprises two integrated modules: a pushback control module and a path planning module. The pushback control module calculates aircraft pushback times and corresponding delay durations under varying taxiway queuing thresholds. Given the large number of feasible taxi routes and the associated computational complexity, the path planning module employs an ant colony optimization algorithm to solve the routing model efficiently.

**Fig 2:**
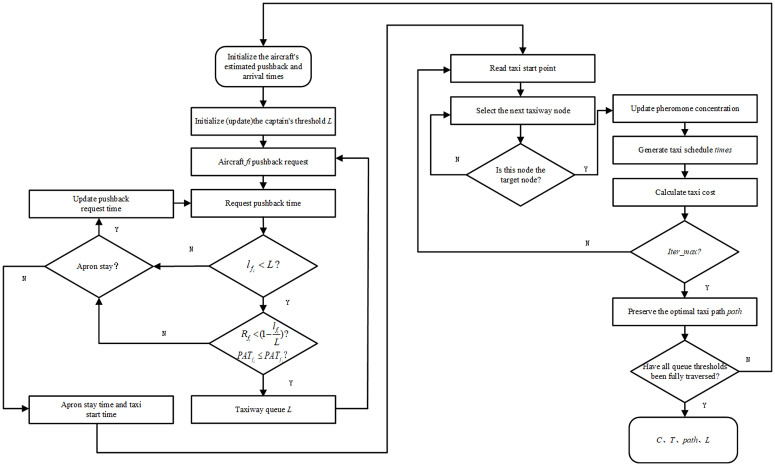
Model-solving flowchart.

To enhance taxiing conflict avoidance during the preplanning stage, the number of potential conflicts with preceding aircraft is quantified and incorporated into the taxi route generation process for each aircraft. This approach enables simultaneous optimization of taxiing costs and conflict constraints. Accordingly, the ant colony optimization algorithm is enhanced by integrating conflict detection and priority discrimination mechanisms into the iterative path optimization process, thereby improving the model’s ability to generate conflict-free taxi routes in the preplanning stage. To further improve computational efficiency, aircraft that have successfully departed are identified along the temporal dimension and removed from the planning queue. The overall solution procedure of the model is illustrated in Fig 2.

**Step 1:** Initialize the planned launch time and approach time, set the maximum queuing threshold, calculate the actual launch time of each departing aircraft under the current queuing threshold, and generate a random number set R to simulate the pushout probability. If the random number is less than the current pushout probability (which is obtained from Eq (1)), then the launch is permitted; otherwise, the launch is rejected. The inbound and outbound aircraft are queued together to form a single queue system. When an aircraft applies for pushback, if the current taxiway captain reaches the set threshold or if the current aircraft pushback time is less than the inbound time of the preceding inbound aircraft, pushback is rejected, and the aircraft remains in the parking space waiting for the next application. The set of actual taxi start slots of incoming and outgoing aircraft is given [AADS,TADS] , and the total delay time $T_{k}^{\text{delay}}(k\in N$ is passed to the next stage.

**Step 2:** The taxi paths are solved separately for incoming and outgoing aircraft according to the number of aircraft movements. The total number of ants is set to $iter_{max}\;$, and the maximum number of iterations is set to. The m ants select the next node in turn according to the distance between nodes and the pheromone concentration and move into their respective taboo table. Tabu until the target node is selected. The length of the current path is calculated and stored in the distance table.

**Step 3:** Update the pheromone concentrations. The length of the path that each ant passes through from the table distances is removed in turn, and if it is less than the current value, the path length and the optimal path best path are updated. The set of allowed paths for the current aircraft is removed; if the current aircraft is the first aircraft, the number of conflicts is set to 0; otherwise, the time for the aircraft on the current path to pass through each node on the path is calculated, and conflict calculations are performed with the optimal paths of the preceding aircraft to compare the priority sizes of the two aircraft, and if the priority of the current aircraft is large, the number of conflicts is not counted. Otherwise, the conflict count $con_{\alpha }^{f_{i}}(k\in N$ is generated. Using the current path length and the number of conflicts as pheromone influencing factors, the pheromone update formula is as follows:


$$\tau_{uv}\{f_{i}\}(n)=(1-\rho )\tau_{uv}\{f_{i}\}(n-1)+\sum_{k=1}^{m}\Delta \tau_{uv}^{k}\;$$ (18)

$$\Delta \tau_{uv}^{k}=\frac{Q}{L^{k}\cdot e^{con^{k}}}\;$$ (19)

**Step 4:** The current iteration number is increased byiter=iter+1 , and the pheromone concentration is updated. If the number of iterations reaches the maximum number of iterations $iter_{max}\;$, the loop ends, keeping the optimal path of the current aircraft to, generating the current aircraft taxi schedule and storing it in *times* and returning to step 2.

**Step 5:** Take the optimal taxi path for each aircraft from *path* and calculate the total taxi distance *minD*. The taxi schedule from *times* is used to calculate the number of conflicts between aircraft again, calculate the penalty time on the basis of the number of conflicts, and convert it to fuel consumption.

**Step 6:** Calculate the current objective function solution; if the glide pending queue threshold is less than the maximum threshold at this point, return to Step 1 until it runs to the maximum threshold. The solution with the smallest objective function is selected as the optimal solution.

## Case study

### Dataset

The experimental dataset consists of 77 aircraft movements recorded during the peak period from 08:00–10:30 on a selected day at the study airport. Representative samples of these data are presented in Table 3, where 0 represents arriving aircraft and 1 represents departing aircraft. The taxi fuel consumption parameters are derived from observations on the same date. Specifically, the fuel cost is set at 1 USD per minute, the conversion coefficient is 0.3734, the penalty factor is 2 minutes, the average taxiing speed is assumed to be 300 m/min, and the minimum tail-flow separation is constrained to 1 minute.

**Table 3:** Aircraft taxiing schedule.

Aircraft number	Original launch time or time of entry	Taxiway starting point	Sliding finish point	Land/Departure	Priority index
1	8:00	24	19	0	1
2	8:01	15	73	1	5
3	8:01	19	73	1	5
4	8:04	24	34	0	2
5	8:05	23	73	1	5
6	8:05	40	73	1	5
7	8:06	49	73	1	5
8	...	...	...	...	...

**Fig 3:**
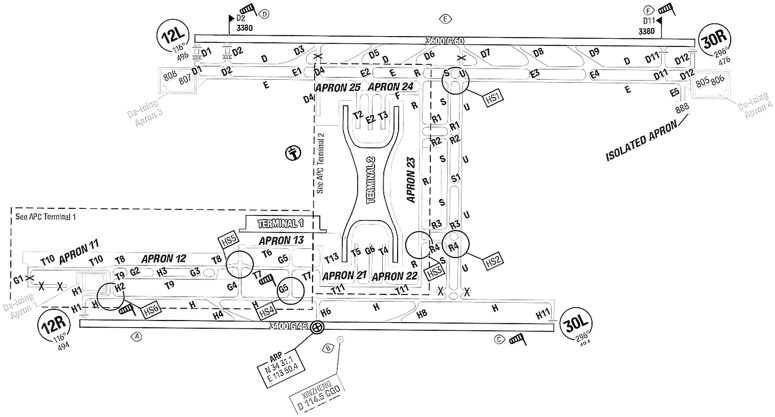
Airport layout plan.

A large airport field taxi system in China is used as an experimental object for simulation, and the plane layout of the airport is shown in Fig 3. Among them, 12R/30L is the main runway with a large amount of flight traffic, so this paper selects flights landing and taking off on this runway for simulation verification. Other layout information is known, including the distance between any two nodes and the number of aprons to which the parking space belongs.

### Analysis of the simulation results

The parameters of the improved ant colony algorithm (IACA) are presented in Table 4.These parameters were selected based on preliminary numerical experiments and established values commonly adopted in studies on airport surface movement optimization. To further assess the robustness of the proposed algorithm, a sensitivity analysis of key algorithmic parameters is conducted in the subsequent subsection.

**Table 4:** IACA algorithm parameters.

Algorithmic parameter	Value
Maximum number of iterations	50
Pheromone weighting	6
Heuristic factor weights	3
Pheromone volatility factor	0.1
Pheromone constant	100

To investigate the impact of algorithm parameters on solution quality, a sensitivity analysis was performed on four key parameters, namely the maximum number of iterations ($iter_{\max}\;$), Pheromone Weight (*α*), Heuristic Factor Weight (*β*), Evaporation Rate (*ρ*), and Pheromone Constant (Q). Each parameter was varied within a reasonable range while keeping the others fixed.

**Fig 4:**
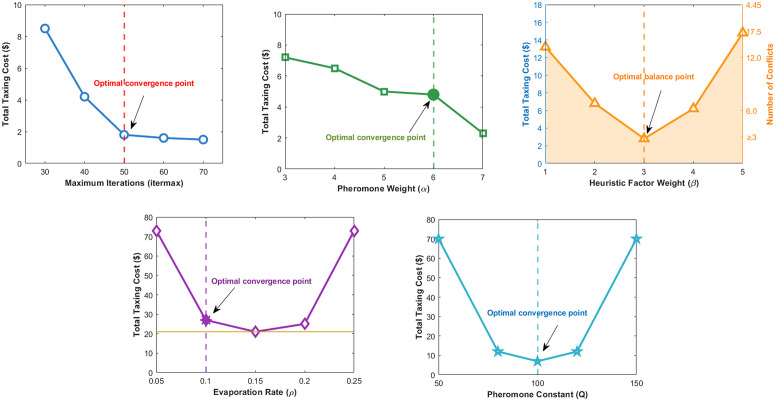
Sensitivity analysis of key parameters in the improved ant colony algorithm.

As shown in Fig 4(a), when $iter_{\max}\;$ is relatively low, the algorithm fails to fully converge, yielding suboptimal solutions. Once $iter_{\max}\;$ reaches approximately 50, further increases yield only marginal improvements in the objective function, indicating near-convergence. Additional iterations beyond this point provide limited performance gains at the expense of increased computational cost. Therefore, $iter_{\max}\;$ = 50 is selected as a balanced and efficient setting.

Fig 4(b) illustrates the influence of the pheromone importance weight (*α*). As *α* increases from 3 to 6, the total cost decreases steadily, suggesting that greater emphasis on historical pheromone trails enhances solution quality. However, excessively high values of *α* may lead to premature convergence due to over-exploitation. Balancing convergence stability and global exploration capability, *α* = 6 is chosen as the optimal value.

As depicted in Fig 4(c), the total cost exhibits a distinct U-shaped trend with respect to the heuristic information weight (*β*). The minimum cost is achieved at *β* = 3, reflecting an optimal balance between heuristic guidance and pheromone influence. Lower values diminish heuristic guidance, while higher values promote greedy behavior, both resulting in degraded solutions.

Fig 4(d) shows the effect of the pheromone evaporation rate (*ρ*). Near-optimal performance is attained when *ρ* = 0.1, which strikes an appropriate balance between retaining valuable historical information and avoiding pheromone stagnation. Both excessively low and high evaporation rates result in higher total costs and poorer performance.

As shown in Fig 4(e), the pheromone deposition constant (Q) significantly affects solution quality. The minimum total cost is obtained at Q = 100, corresponding to an appropriate pheromone reinforcement intensity. Values that are either too low or too high lead to unstable path selection and inferior solutions.

#### Results of the pushback strategy.

A comparative analysis is performed between the no-control strategy—in which aircraft join the taxiway queue immediately upon submission of a pushback request—and the proposed DPSC control strategy. The primary operational outcomes of the two strategies are summarized in Table 5.

**Table 5:** Comparison between DPSC and uncontrolled strategies.

Threshold	C(USD$	G(min	$G_{max}(min$	$T_{taxi}(min$	*Gap*
No control	9931.72	–	–	7.17	–
1	8408.33	2.98	9	5.88	15.34%
2	8369.81	2.98	9	5.84	15.73%
3	8198.41	1.75	6	5.82	17.45%
4	8094.34	0.85	3	5.84	17.68%
5	8163.44	0.85	3	5.83	17.80%
6	8228.72	0.85	3	5.88	17.15%
7	8370.54	0.85	3	5.98	15.72%
8	8259.89	0.68	2	5.91	16.83%
9	8197.06	0.32	2	5.87	17.47%
10	8091.51	0.32	2	5.92	16.72%

As shown in Table 5, *G* the average waiting time for a parking space is indicated. $G_{max}\;$ is the maximum waiting time for a parking space, $T_{\text{taxi}}\;$ is the average taxi time. and is the percentage of cost reduction using the DPSC control strategy compared with the no pushback control strategy.

Overall, the aircraft fuel cost decreases with increasing captain threshold and decreases the most when the taxiway threshold is equal to 3, which indicates that when the taxiway queuing threshold is too small, more aircraft are stranded in the parking space for too long, and the overall stay cost is higher. When the taxiway queuing threshold exceeds 3, the aircraft fuel cost tends to be stable and reaches the optimal value when the queuing threshold is equal to 5, and the optimal cost is 8163.44. Under a certain scale of traffic volume, the maximum taxiway queuing threshold is not an optimal solution, and the appropriate queuing threshold can meet the operational needs of the airport.

In addition, with the increase in the queuing threshold, the average waiting time of the aircraft in the parking space *G* maximum dwell time $G_{max}\;$ gradually decreases but not linearly but decreases step wisely, indicating that under a certain queuing threshold, the flight launching slot is less affected by the queuing threshold, and the time affluence is greater, which can be flexibly adjusted according to the actual requirements of a certain range of launching slots.

#### Result analysis based on conflict negative feedback.

**Fig 5:**
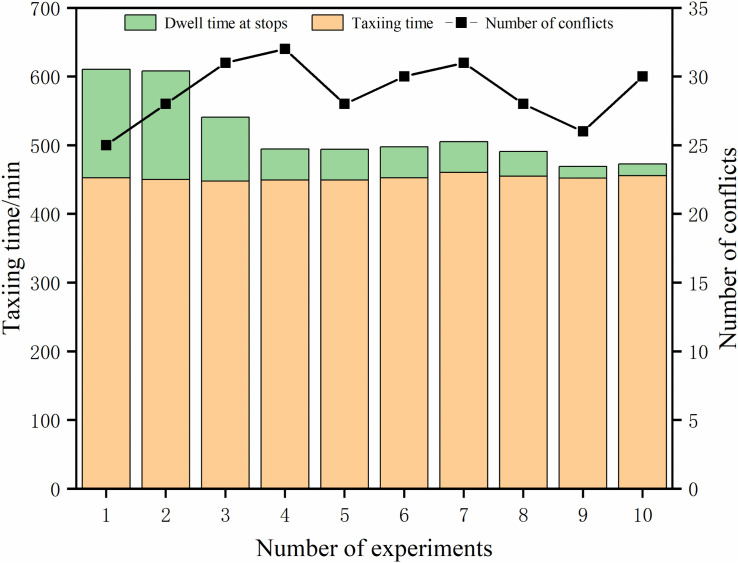
Aircraft taxi and parking wait times.

As shown in Fig 5, for aircraft taxiing and parking bay waiting times, the total aircraft taxiing time decreases as the taxiway queuing threshold increases.

However, the stopping time gradually decreases from a large initial value, indicating that many aircraft wait in the stopping space because they do not meet the pushback requirements when the taxiway queuing threshold is small. At this time, the parking space dwell time becomes the main factor affecting the total cost of aircraft operation, and as the queuing threshold increases further, the aircraft parking space dwell time tends to stabilize, and the interaircraft taxiing conflict becomes the main factor affecting the total cost.

The total cost reaches the minimum when the queuing threshold is equal to 5, but the number of conflicts is not the minimum at this time, which indicates that the excessive pursuit of taxiing in a conflict-free manner does not lead to an optimal value.

Compared with the conflict-free control constraint algorithm, the two algorithms achieve the optimal solution when the threshold of the taxiway is equal to 5 and 7, and the comparison table is shown in Table 6. In Table 6, $T_{\text{taxi}}^{*}\;$ is the average flight taxi time per threshold, ${\text{conf}}^{*}\;$ is the average aircraft taxi cost, and conf  is the average number of flight conflicts.

**Table 6:** Comparison with or without conflict constraint.

Arithmetic	1	2	3
Conflict control	452.39	8264.37	28.9
No control	450.55	9674.68	41.7
Difference	1.84	−1410.31	−12.8

The fuel consumption cost of the algorithm with the conflict constraint module in the updating pheromone phase is reduced by $1412.71 on average compared with that of the unconstrained module, the average taxiing time per threshold of the aircraft is increased by 1.71 min, and the average number of conflicts is reduced by 12.8 times. By improving the ant colony algorithm, imposing conflict constraints and priorities and constraints on aircraft in the preplanning stage effectively reduces the cost of aircraft taxiing, reduces taxiing conflicts, and improves operational efficiency.

**Fig 6:**
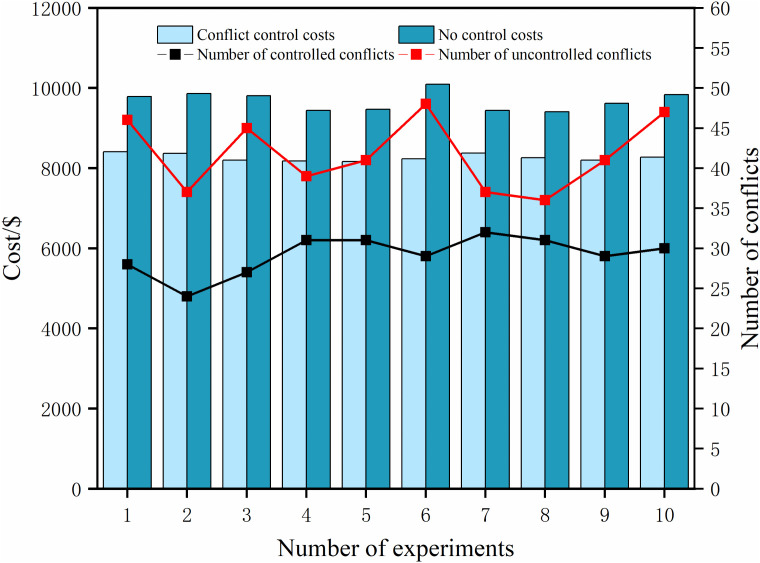
Comparison of fuel costs with and without conflict constraints.

**Fig 7:**
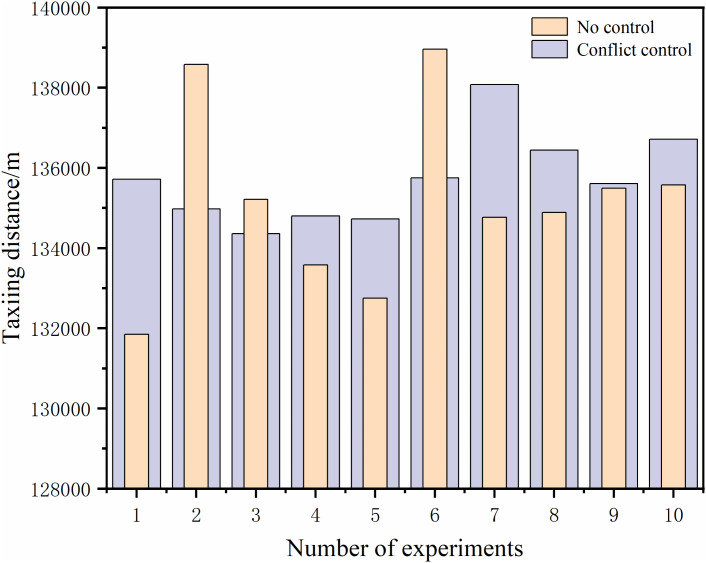
Flight taxiing distance comparison.

Conflict avoidance by aircraft can be achieved in the spatial dimension by changing paths for obstacle avoidance purposes, in addition to avoiding possible conflicts by adjusting the glide onset slot. However, changing paths usually implies a longer glide path, which is well corroborated in our results. The flight taxiing cost and the number of conflicts under each threshold are shown in Fig 6. Compared with the conflict-free constraint, the aircraft taxiing distance pair is shown in Fig 7, and the flight taxiing route with the conflict constraint is longer than the conflict-free constraint under most of the queuing thresholds, except for thresholds 2, 3, and 6.

However, the fuel consumption cost of the conflict-free constraint is always higher than that of the conflict-free constraint, which shows that in the case of the determination of the taxiing gate and runway waiting point, in the case where the taxi gate and the runway waiting point are determined, the use of the conflict-constrained taxiing strategy can reduce the number of taxiing conflicts by changing the paths when the taxiing distance permits and further reduce fuel consumption.

**Fig 8:**
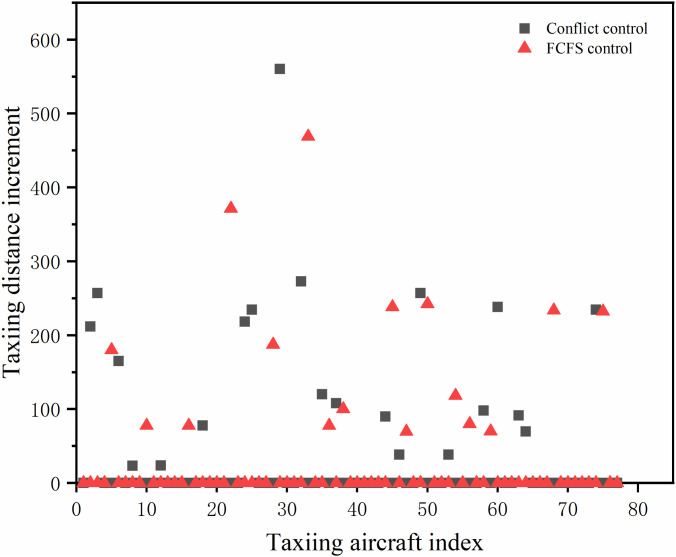
Aircraft taxi and parking wait times.

Compared with the first-come-first-served taxi increment, the conflicting taxi increment has more growth and a larger range of variation, as shown in Fig 8. The total taxi distance increment under the first-come-first-served strategy is 2824.6 m, among which 6 aircraft taxiing increments are more than 200 m. The total taxi distance increment under the conflicting control strategy is 3426.9 m, which is increased by 602.3m compared with that of the first-come-first-served strategy. Under the two different strategies, the vast majority of the aircraft select the shortest path to taxi, and a small number choose the longest way round to avoid the taxiing conflict. A small number of aircraft choose to detour far away to avoid taxiing conflicts, and there are more detours with conflict control constraints than with the first-come-first-served strategy does, and the effect of route switching is obvious.

## Conclusions

This results in considerable unnecessary fuel consumption and substantially increased operational costs for airlines. In the preplanning stage of aircraft taxiing, regulating pushback frequency and mitigating potential conflicts during actual taxi operations not only reduce the workload of air traffic controllers but also enhance the overall safety of airport surface operations. To address these challenges, this study proposes a taxi path planning method based on a dynamic pushback slot control (DPSC) strategy, which aims to determine optimal taxi routes for multiple aircraft under varying taxiway queuing thresholds. The main contributions and findings of this work are summarized as follows.

1. Considering realistic airport operational characteristics, a dynamic pushback control strategy is developed in which pushback frequency is adjusted in real time according to the current taxiway queue length. A cost conversion function is introduced to translate gate waiting time into equivalent fuel consumption costs, and a coordinated queuing mechanism for inbound and outbound aircraft is established. This mechanism improves taxiway resource utilization while reducing fuel consumption by optimizing the pushback timing of departing aircraft.
2. Under practical operational constraints, an objective function integrating aircraft taxiing time and gate waiting time is formulated, and a two-stage optimization framework for taxi path planning is constructed. To enhance conflict avoidance efficiency, an aircraft priority scheduling queue is designed based on priority dispatching principles. Furthermore, an improved ant colony optimization algorithm incorporating conflict detection is proposed to solve the resulting optimization problem.
3. Aircraft operating costs are governed by a trade-off among parking dwell time, taxiing conflicts, and taxiing duration. By setting appropriate taxiway queuing thresholds, fuel consumption can be effectively reduced while improving taxiway operational efficiency. Experimental results demonstrate that, compared with an uncontrolled strategy, the proposed approach reduces taxiing costs by 17.8%, achieving an optimal cost of $8,163.44, thereby validating the effectiveness of the proposed optimization model.
4. Since this paper treats the inbound aircraft landing time as the taxi start time and ignores the effect of the inbound time on the model perturbation, future research should focus on the taxi route assignment scheme under the inbound time error. In addition, future research should focus on modeling aircraft taxi route assignment in the multirunway case.

Further numerical analyses reveal that aircraft operating costs are jointly influenced by parking dwell time, taxiing time, and the number of taxiing conflicts. This indicates that optimizing a single performance indicator—such as taxiing distance or conflict count—does not necessarily yield optimal operational outcomes. In practice, identifying suitable taxiway queuing thresholds enables airports to balance efficiency and safety objectives, particularly during peak departure periods.

Despite these advantages, several limitations of the present study should be acknowledged. First, inbound aircraft arrival times are assumed to be deterministic and are directly treated as taxi start times, whereas arrival time uncertainty may significantly affect taxiway congestion and conflict propagation. Second, the proposed framework is evaluated under a single-runway configuration, and its applicability to multi-runway and mixed-mode operations remains to be explored. In addition, fixed gate assignments and runway holding positions are assumed, while dynamic gate reassignment may further influence taxiing behavior.

Future research will focus on incorporating arrival time uncertainty into the taxi route planning framework, extending the proposed strategy to multi-runway airport environments, and integrating real-time operational data to enhance the adaptability and robustness of the control strategy. These efforts are expected to further improve the practical applicability of the proposed method.

## Supporting information

S1 Data Dataset. (ZIP)

## References

[1] Cao F, Tang T-Q, Gao Y, You F, Zhang J. Calculation and analysis of new taxiing methods on aircraft fuel consumption and pollutant emissions. Energy. 2023;277:127618. doi: 10.1016/j.energy.2023.127618

[2] Kharoufah H, Murray J, Baxter G, Wild G, Fernandes H, Müller C. A review of human factors causing commercial air transport accidents and incidents: 2000–2016. Prog Aerosp Sci. 2018;99:1–13. doi: 10.1016/j.paerosci.2018.03.002

[3] Rathinam S, Montoya J, Jung Y. An optimization model for reducing aircraft taxi times at the Dallas Fort Worth International Airport. In: Proceedings of the 26th International Congress of the Aeronautical Sciences (ICAS). Anchorage, Alaska, USA: International Council of the Aeronautical Sciences; 2008. Available from: doi: 10.1016/j.jairtraman.2021.102077

[4] Deau R, Gotteland JB, Durand N. Airport surface management and runways scheduling. In: 8th USA/Europe Air Traffic Management Research and Development Seminar (ATM2009). Napa, California, USA: FAA/Eurocontrol; 2009.

[5] Roling PC, Visser HG. Optimal Airport Surface Traffic Planning Using Mixed-Integer Linear Programming. Int J Aerospace Eng. 2008;2008:1–11. doi: 10.1155/2008/732828

[6] Anderson R, Milutinović D. An approach to optimization of airport taxiway scheduling and traversal under uncertainty. Proc Instit Mech Eng Part G J Aerospace Eng. 2012;227(2):273–84. doi: 10.1177/0954410011433238

[7] Guépet J, Briant O, Gayon JP, Acuna-Agost R. The aircraft ground routing problem: Analysis of industry punctuality indicators in a sustainable perspective. Eur J Operat Res. 2016;248(3):827–39. doi: 10.1016/j.ejor.2015.08.041

[8] Park DK, Kim JK. Influential factors to aircraft taxi time in airport. J Air Transp Manag. 2023;106:102321. doi: 10.1016/j.jairtraman.2022.102321

[9] Soltani M, Ahmadi S, Akgunduz A, Bhuiyan N. An eco-friendly aircraft taxiing approach with collision and conflict avoidance. Transp Res Part C Emerg Technol. 2020;121:102872. doi: 10.1016/j.trc.2020.102872

[10] Gao Y, Tang T-Q, Cao F, Zhang J, Wang R. A two-phase total optimization on aircraft stand assignment and tow-tractor routing considering energy-saving and attributes. Sustain Energy Technol Assess. 2023;57:103237. doi: 10.1016/j.seta.2023.103237

[11] Yan J, Hu H, Wang Y, Ma X, Hu M, Delahaye D, et al. Robust pre-departure scheduling for a nation-wide air traffic flow management. Chin J Aeronaut. 2025;38(4):103223. doi: 10.1016/j.cja.2024.08.054

[12] Fan X, Wang M, Wang Y, Hu R. Equity and efficiency trade-off in allocating airport and airspace capacity in a multiple airport system. Transp Res Part A Policy Pract. 2025;200:104645. doi: 10.1016/j.tra.2025.104645

[13] Weiszer M, Burke EK, Chen J. Multi-objective routing and scheduling for airport ground movement. Transp Res Part C Emerg Technol. 2020;119:102734. doi: 10.1016/j.trc.2020.102734

[14] Maadanpour Safari F, Etebari F, Pourghader Chobar A, Lotfian Delouyi A, Safaeian M, Babapour Azar A. Modelling and optimization of a tri-objective transportation-location-routing problem considering route reliability: using MOGWO, MOPSO, MOWCA and NSGA-II. J Optim Indust Eng. 2021;14(2):83–98. doi: 10.22094/JOIE.2020.1893849.1730

[15] Brownlee AEI, Weiszer M, Chen J, Ravizza S, Woodward JR, Burke EK. A fuzzy approach to addressing uncertainty in Airport Ground Movement optimisation. Transp Res Part C Emerg Technol. 2018;92:150–75. doi: 10.1016/j.trc.2018.04.020

[16] Sekine K, Kato F, Tatsukawa T, Fujii K, Itoh E. Rule Design for Interpretable En Route Arrival Management via Runway-Flow and Inter-Aircraft Control. IEEE Access. 2023;11:75093–111. doi: 10.1109/access.2023.3297136

[17] Benlic U, Brownlee AEI, Burke EK. Heuristic search for the coupled runway sequencing and taxiway routing problem. Transp Res Part C Emerg Technol. 2016;71:333–55. doi: 10.1016/j.trc.2016.08.004

[18] Katsigiannis FA, Zografos KG, Fairbrother J. Modelling and solving the airport slot-scheduling problem with multi-objective, multi-level considerations. Transp Res Part C Emerg Technol. 2021;124:102914. doi: 10.1016/j.trc.2020.102914

[19] Ahmadian MM, Salehipour A. Heuristics for flights arrival scheduling at airports. Int Trans Operat Res. 2020;29(4):2316–45. doi: 10.1111/itor.12901

[20] Idrissi O, Bikir A, Mansouri K. Efficient Management of Aircraft Taxiing Phase by Adjusting Speed Through Conflict-Free Routes. Stat Optim Inf Comput. 2022;10(1):12–24. doi: 10.19139/soic-2310-5070-1162

[21] Ravizza S, Atkin JAD, Burke EK. A more realistic approach for airport ground movement optimisation with stand holding. J Sched. 2013;17(5):507–20. doi: 10.1007/s10951-013-0323-3

[22] Goncharenko VI, Lebedev GN, Martynkevich DS, Rumakina AV. Operational planning of aircraft routes when servicing a random stream of requests arriving during the flight. J Phys Conf Ser. 2021;1958(1):012016. doi: 10.1088/1742-6596/1958/1/012016

[23] Feuser Fernandes H, Müller C. Optimization of the waiting time and makespan in aircraft departures: A real time non-iterative sequencing model. J Air Transp Manag. 2019;79:101686. doi: 10.1016/j.jairtraman.2019.101686

[24] Bao J, Kang J, Zhang J, Zhang Z, Han J. A dynamic control method for airport ground movement optimization considering adaptive traffic situation and data-driven conflict priority. J Air Transp Manag. 2025;124:102753. doi: 10.1016/j.jairtraman.2025.102753

[25] Wang Z, Wang Y, Xu W, Wang H, Liu F. A data-driven approach for determining airport declared capacity. Transp Res Part C Emerg Technol. 2025;171:105012. doi: 10.1016/j.trc.2025.105012

[26] Desai J, Lian G, Srivathsan S. Dynamic departure pushback control at airports: Part A—Linear penalty‐based algorithms and policies. Naval Res Logist. 2024;71(7):960–75. doi: 10.1002/nav.22189

[27] Wang Y, Liu C, Wang H, Duong V. Slot allocation for a multiple-airport system considering airspace capacity and flying time uncertainty. Transp Res Part C Emerg Technol. 2023;153:104185. doi: 10.1016/j.trc.2023.104185

